# Efficacy of an interdental brush in cleaning artificial plaque on a 3D-printed model base

**DOI:** 10.1186/s12903-022-02451-4

**Published:** 2022-09-22

**Authors:** Seo Eun Kim, Eun Sun Song, Seung Pyo Lee

**Affiliations:** Department of Oral Anatomy, Dental Research Institute, School of Dentistry, Seoul National University, Seoul, Korea

**Keywords:** Interdental brush, Plaque removal, 3D-printed model, Gingivitis, Periodontal diseases

## Abstract

**Background:**

Among interdental cleaning aids (ICAs), interdental brushes (IDBs) are in the spotlight because they can effectively remove plaque from interdental surfaces. Guidance on the correct use of ICAs, such as IDBs, is required to prevent dental plaque accumulation. Since it is impossible to confirm the interdental proximal surface unless extracted, it is difficult to conduct quantitative experiments. This study presented an efficient way to evaluate IDBs by realizing dental structures and embrasures using a Dental computer-aided design (CAD) software and a 3D printer.

**Methods:**

Two different sizes of embrasure (0.7 and 1.2 mm) crown models were prepared with CAD software and a 3D printer. To evaluate the cleaning efficacy of IDBs of each size (0.6, 0.7, 0.8, 1.0, 1.2, and 1.5 mm diameters), the 9th cycle of brush move was performed where artificial plaque was spread and a digital camera was used to record the process. The pixels and percentage of cleaning from the recorded digital images were analyzed.

**Results:**

The plateau was formed after the 5th brushing cycle under all conditions—after the 5th cycle, the cleaning efficacy of the two crown models was 69.3–86.4% and 49.8–75.4%. In these results, the optimal diameters for the IDB were 1.2 and 1.5 mm for embrasure sizes of 0.7 and 1.2 mm, respectively. Moreover, the cleaning efficacy was the highest at 86.4% and 75.4% after the 9th cycle.

**Conclusions:**

The 3D-printed model base for the human oral embrasure structure is an adequate model to test artificial plaque removal using IDB. The use of IDBs for more than five cycles does not support the conventional idea that a greater number of IDB brushing moves is more effective in a statistically substantial manner.

## Background

Dental plaque is known to be one of the main reasons causing gingivitis and periodontal diseases [1]. Despite the conventional use of a toothbrush to regulate the amount of plaque, there has been growing public and academic interest regarding the use of interdental cleaning aids (ICAs) conjointly [2–5]. Among the currently available ICAs, IDBs are of particular significance since IDBs have been found to show efficacy compatible with or higher than floss or other aids [3, 6, 7]. There was a systematic review that the use of IDB showed superior scores in comparative plaque index, mean probing depth, relative interdental papillae level, and more, especially the higher bleeding index for IDB [2]. And randomized controlled trial described the different efficacy between the use of floss and IDB in 55 subjects and concluded that the removal of interdental plaque was better in persons who used the IDB [8].

In current therapeutic practice, there is only a conventional guideline for patients to use an IDB that is slightly greater than their interdental gap [9]. This leads to the need for an investigation of the correlation between the size of the IDB and embrasure on a practical model. There has been a clinical attempt to investigate the influence of the shape of the IDB on its cleaning efficacy. However, a technical limitation was imposed that only the interdental space on the buccal side could be investigated [10]. Similarly, a study investigated geometrical and anatomical models with interdental spaces ranging from 1 to 4 mm [11] whereas the actual embrasure is sized at approximately 0.4–0.5 mm in the human oral structure.

To acquire three-dimensional information on complex structures, such as human maxillary and mandibular teeth, and jaw structures, Dental computer-aided design/computer-aided manufacturing (CAD/CAM) and 3D printing systems have been used in various dental fields lately—endodontics, oral and maxillofacial surgery, and prosthodontics [12–15]. For instance, when reconstructing a damaged mandible, 3D-surgical modeling and fabrication are enabled by a 3D printer with high precision and reproducibility. In prosthodontics, particularly, a combination of CAD/CAM and 3D printing is highlighted over the conventionally used lost-wax casting with crowns and bridges, for improved manufacturing time and precision [12, 13, 16–18].

Throughout the literature, precedent research using a 3D-printed model was found to prepare morphologically equivalent pairs of embrasure structures (1.0, 1.1, and 1.3 mm) to testify the artificial plaque removal efficacy of variously sized IDBs [19]. However, the preceding studies, including the aforementioned model studies, were unable to mimic the true embrasure structure adequately, which led to the pursuit of a standardized model, such as a 3D-printed model, to understand the intrinsic plaque removal performance of the IDB. Here we report an improved mimicking of a dental structure and embrasure using a 3D printer and relevant 3D software, where the CAD program enables an unrestrained structural and spatial adjustment of the interdental environment by parameter alterations, followed by a 3D printing of the architecture in a rapid, easily accessible manner. A plaque removal examination was performed with IDBs of different sizes, to identify the optimal diameter for the given embrasure gap.

## Methods

### Materials

**Fig. 1:**
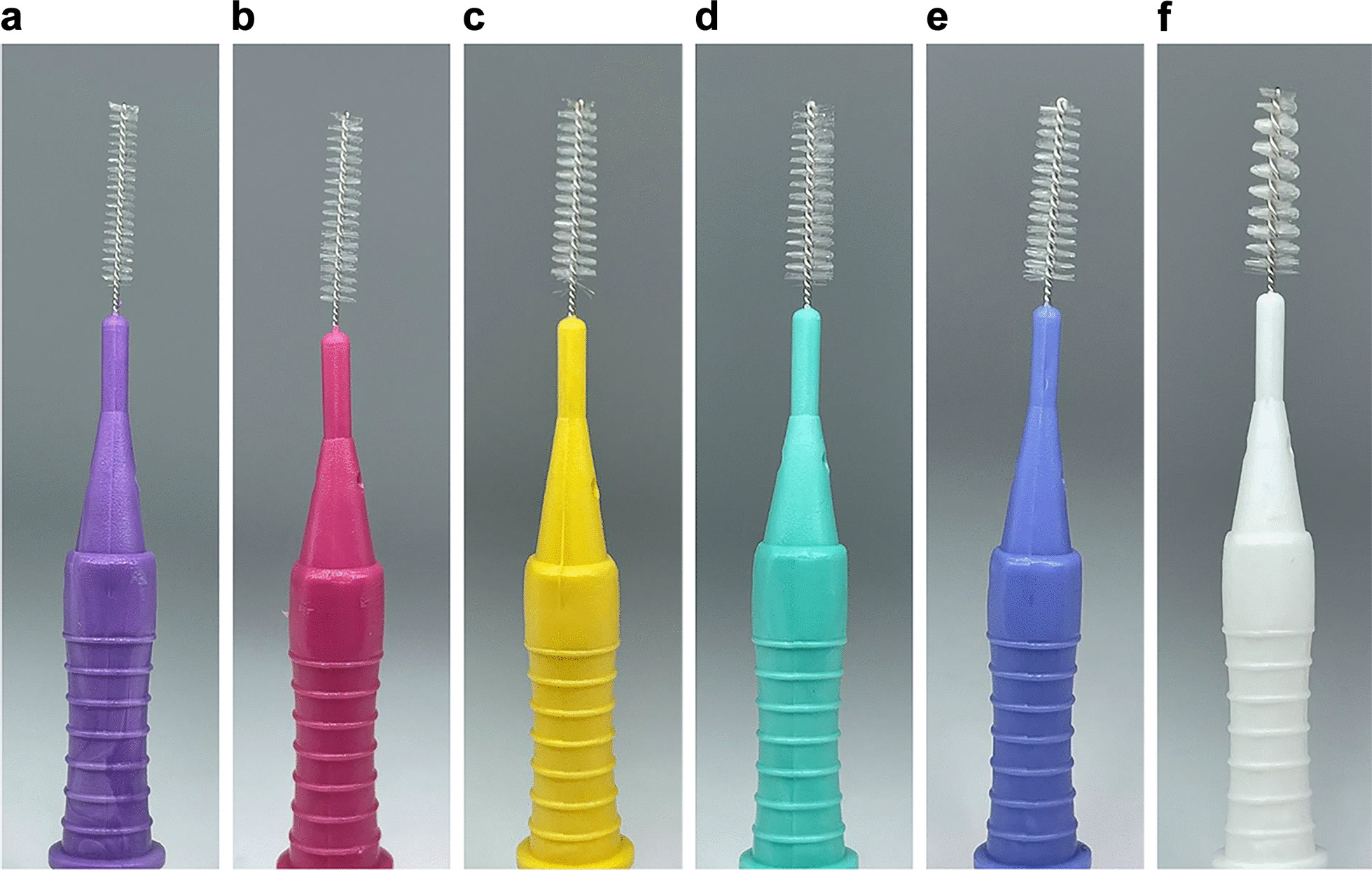
Front profiles of the six IDBs examined in this study: **a** 0.6 mm diameter, **b** 0.7 mm diameter, **c** 0.8 mm diameter, **d** 1.0 mm diameter, **e** 1.2 mm diameter, and **f** 1.5 mm diameter

A 3D-printing resin (A2 Permanent, One Digital System Co., Ltd., Korea) was used to prepare crowns and model bases by the 3D printer which has Knoop hardness number-HK enamel 350 and A2 permanent 317. Artificial dental plaque (Artificial Plaque, Nissin Dental Products Inc., Japan) was used. Figure 1 shows cylindrical IDBs with diameters of 0.6, 0.7, 0.8, 1.0, 1.2, and 1.5 mm (I type, D-all, Korea).

### Model preparation

**Fig. 2:**
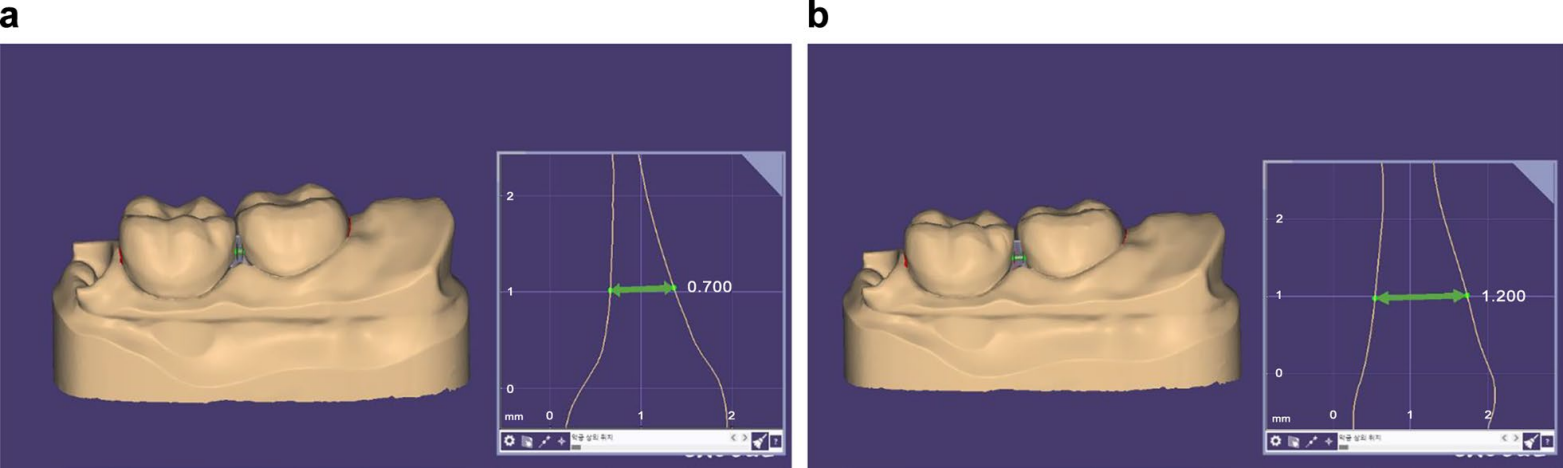
Representative digital images of **a** 0.7 mm embrasure and **b** 1.2 mm embrasure with cross-sectional images in the square

**Fig. 3:**
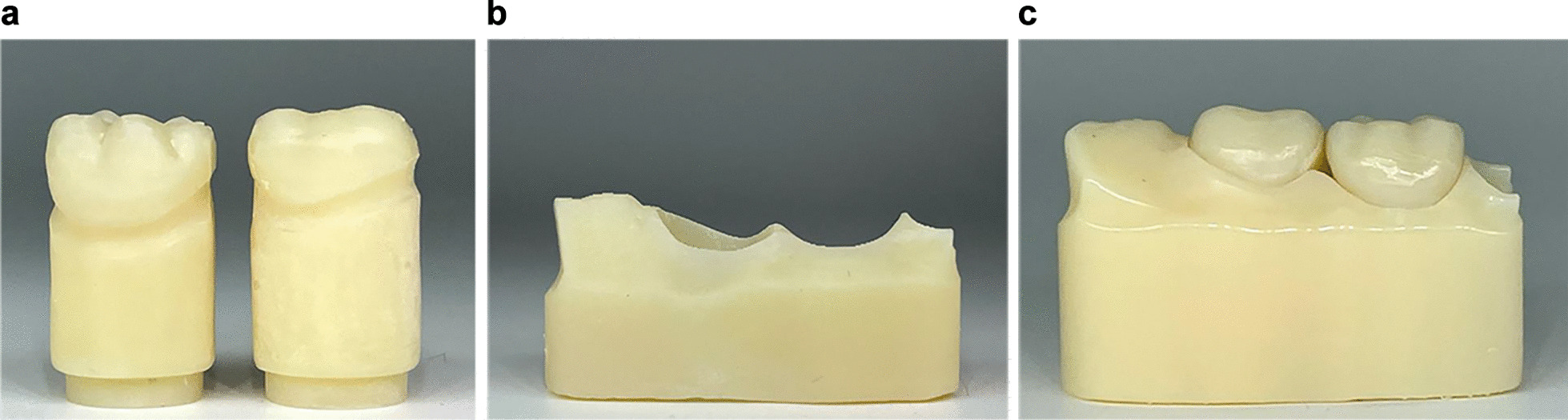
Representing images of the 3D-reproduced crown dye and model base for IDBs tested. **a** Model, **b** Modelbase, **c** Combined model and modelbase

The detailed model preparation procedure is described as follows. (1) Model design: Dentiform #46 and 47 (3D-scanner, Medit., Korea) were scanned to be processed on the CAD program (Exocad Dental CAD, Exocad GmbH, Darmstadt, Germany) to design the models with embrasures of 0.7 and 1.2 mm (Fig. 2). (Model type: plate-less model with cutout dies) (2) 3D printing of the model: Given the STL file of the designed dye model, the connector and base were formed via Chitubox program on the 3D-printer (Ka:rv LP-600, Shinwon Dental, Korea), followed by a slicing procedure and conversion into a CTB file. The CTB file was used as the input on the 3D printer, where A2 Permanent Resin was filled in the 3D printer tank to print the dye model. (3) Surface cleaning: 98% ethanol was used for 10 min to remove residual impurities from the surface of the printed dye model. (4) Model hardening: The cleaned dye model was exposed to a 300 W UV LED light in the Curebox (One Digital System Co., Ltd., Korea) for 7 min followed by 3 min of cooling. (5) Post-treatment: Connectors were removed from the hardened dye model by scissors or a nipper, followed by surface polishing with a Denture Pad or Rubber Point (Fig. 3). The detailed printing parameters are as follows. Layer height: 0.1 mm; bottom layer count: 3; exposure time: 5.0 s; bottom exposure time: 35.0 s; transition layer count: 5; transition type: linear; transition time decrement: 5.0 s; waiting mode during printing: resting time; Rest time before lift: 0.0 s; rest time after lift: 0.0 s; rest time after retract: 0.5 s; bottom lift distance: 2.0 + 3.0 mm; lifting distance: 1.0 + 2.0 mm; bottom retract distance: 4.0 + 1.0 mm; retract distance: 2.0 + 1.0 mm; bottom lift speed: 60.0 and 100.0 mm/min; lifting speed: 150.0 and 300.0 mm/min; bottom retract speed: 100.0 and 60.0 mm/min; retract speed: 300.0 and 100.0 mm/min.

### Sample preparation and experimental method

Crown material was evenly spread on the distal surface of the first molar and the mesial surface of the second molar, followed by air-drying over 30 min. The artificial plaque was evenly spread in the same manner, followed by air-drying over 30 min [19]. For an accurate moving test, a model base (gum) was manufactured so that teeth could be inserted and removed and controlled. The model itself was fixed, elbow rest was given at a certain position, and positioning was performed so that only the reciprocating motion could be performed. In addition, the same test was performed from buccal to lingual. The experiment was conducted to ensure repeatability in consideration of the use of IDB in the buccolingual direction. These procedures were performed by a professionally trained dental hygienist. In total, nine cycles of the move were performed since the cleaning efficacy reached a plateau state and converged to final efficacy. A digital camera was used to record the images of the 3D-printed crown dye for each cycle.

### Efficacy assessment

**Fig. 4:**
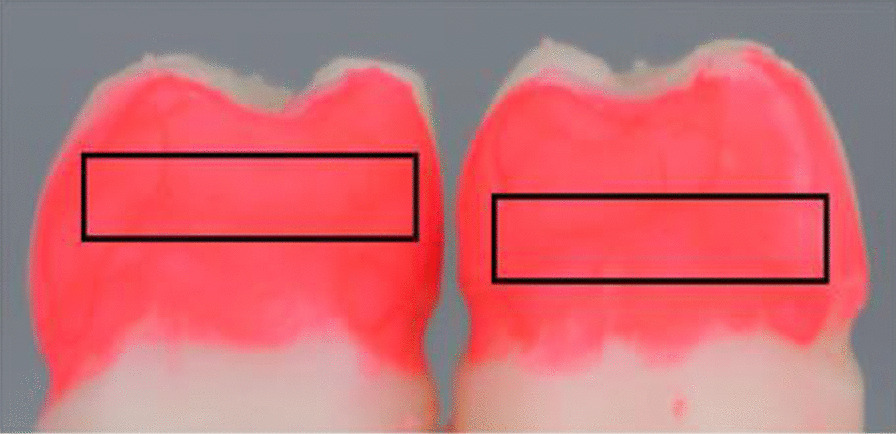
A representative image of a rectangle area of 2.08 cm × 0.47 cm was set from the bottom part of the point of contact between the first molar (distal surface) and the second molar (mesial surface)

To evaluate the cleaning efficacy of IDBs of each size, a digital camera (EOS M100, Canon Inc., Japan) was used to record the images of embrasure before and after the brushing. A rectangle area of 2.08 cm × 0.47 cm was set from the bottom part of a point of contact between the first molar (distal surface) and the second molar (mesial surface) of the lower jaw (Fig. 4), where the remaining amount of the artificial plaque was measured after the brushing. We used the wand tool (tracing tool) of ImageJ (U.S. National Institute of Health, Bethesda, Maryland, USA). After setting the correct area of 2.08 cm × 0.47 cm set by 0% with the artificial plaque applied to the entire tooth, the erased part per cycle to evaluate the pixels and percentage of cleaning concerning the recorded digital images.

## Results

**Fig. 5:**
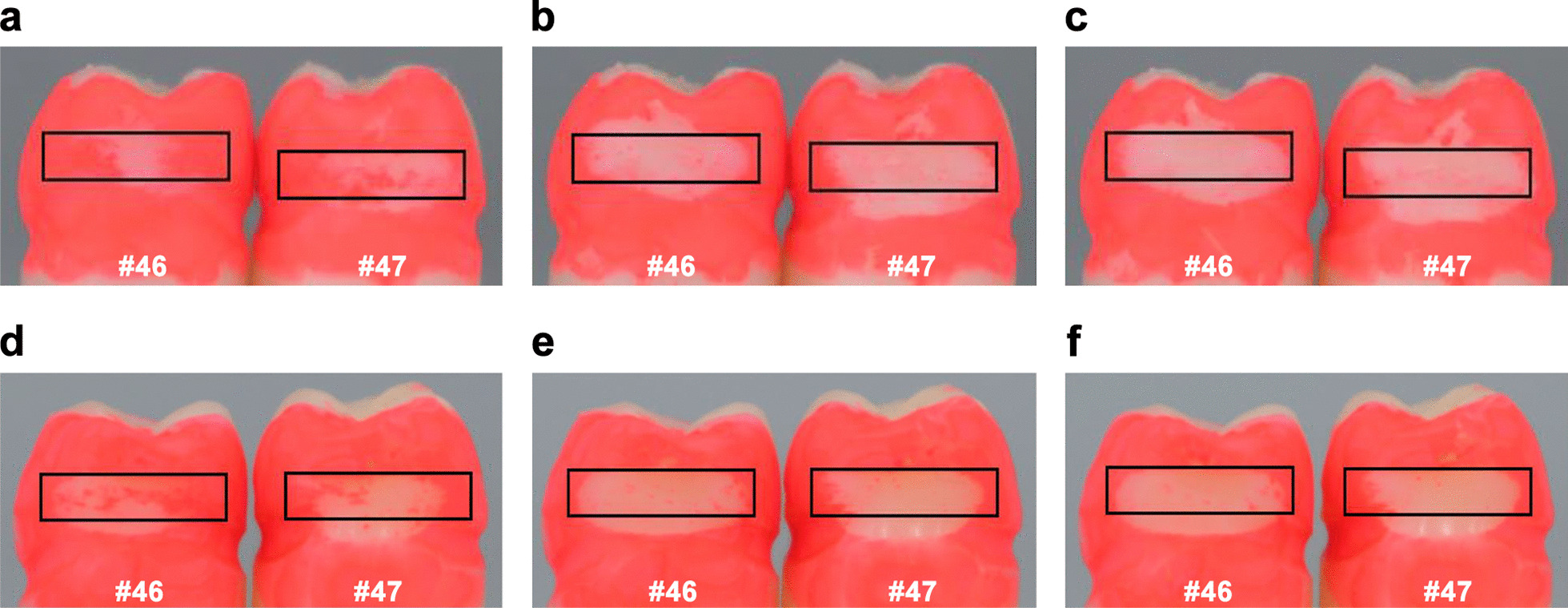
Representative digital images of the artificial plaque-coated #46 distal surface and the #47 mesial surface (0.7 mm embrasure) after **a** 1st, **b** 5th, and **c** 9th brushing cycle with the 0.7 mm IDB, and **d** 1st, **e** 5th, and **f** 9th brushing cycle with the 1.2 mm IDB

**Fig. 6:**
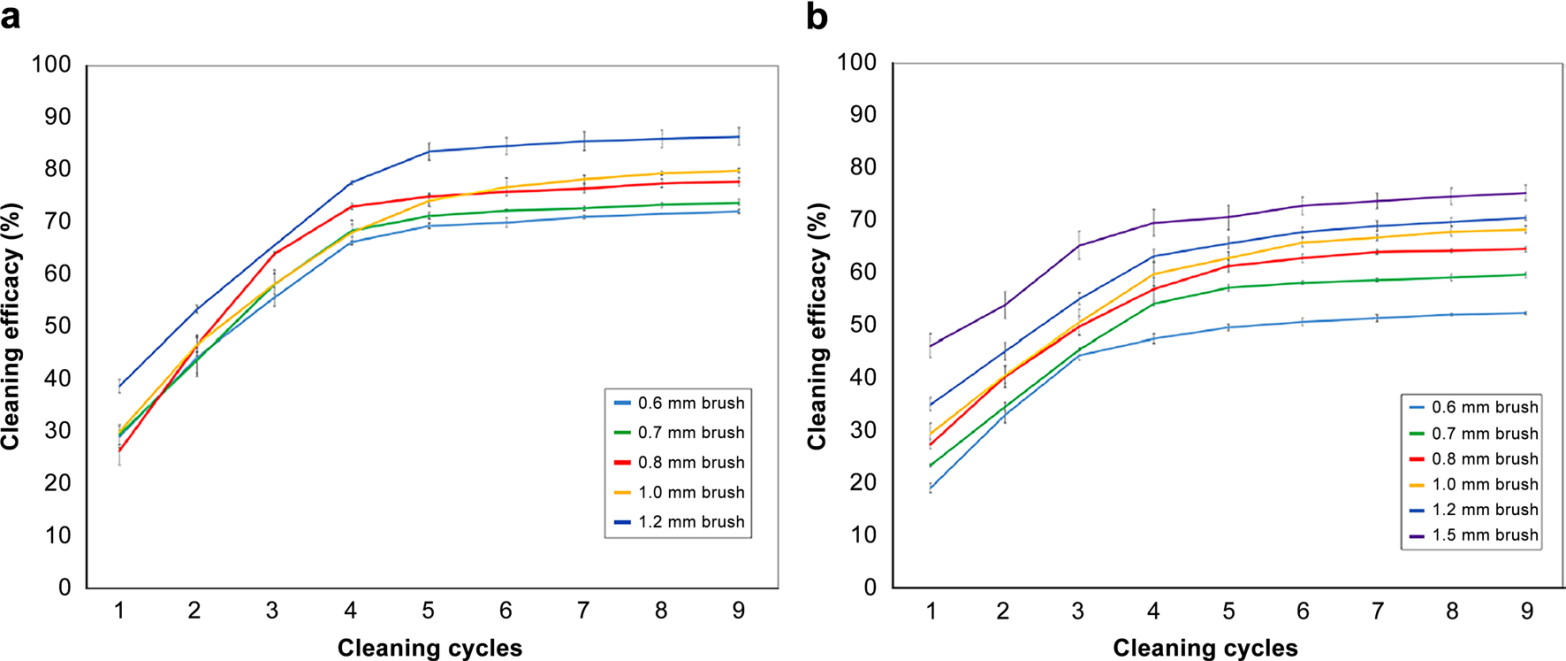
Gradual increment and convergence of the cleaning efficacy over the cleaning cycles at **a** 0.7 mm embrasure and **b** 1.2 mm embrasure. (Error bar: mean error; five repetitions for each data point)

First, we embarked on the design and manufacturing of the model base with two different embrasure sizes of 0.7 and 1.2 mm. IDBs of sizes of 0.6-, 0.7-, 0.8-, 1.0-, and 1.2-mm diameter were examined for up to nine cycles to remove the artificial plaque between the #46 distal surface and the #47 mesial surface. As shown in Fig. 5a–c, digital images of the surfaces (0.7-mm embrasure) were recorded after the 1st, 5th, and 9th cleaning cycles, where the numbers of pixels in the black rectangles were counted by ImageJ to estimate the cleaning efficacy in percentage. In the case of the 1.2 mm embrasure model, an extra IDB with a diameter of 1.5 mm was used besides the five aforementioned IDBs. Similar to the case of 0.7 mm embrasure, the same demonstration protocol was conducted with the 1.2 mm embrasure model base, which resulted in a similar consequence (Fig. 5d–f). As shown in Fig. 6a, the cleaning efficacy gradually improved from 0 to 5 cycles. The starting efficacy in the 1st cycle was only between 28.9 and 38.6%, whereas that in the 5th cycle was between 69.3 and 83.5%. However, the efficacy reached a plateau state at the 5th cycle and showed a converging trend to the efficacy range between 72.0 and 86.4% at the 9th cycle where we stopped further repetition. Moreover, as shown in Fig. 6b, the removal efficacy based on the counted pixels continuously improved from 0 to 5 cycles. The starting efficacy in the 1st cycle was only between 19.3 and 46.3% whereas that in the 5th cycle was between 49.8 and 70.7%. Similarly, the cleaning efficacy converged to a range between 52.5 and 75.4%. To summarize, the IDB with a diameter of 1.2 and 1.5 mm was found to be the optimal size for 0.7 and 1.2 mm embrasures, respectively (Tables 1, 2).

**Table 1:** Summary of standard cleaned area, cleaning efficacy, and standard error for interdental brushes of five sizes over the cycles from 0 to 9 at 0.7 mm embrasure (n = 5)

Brush stem diameter (mm)	0.6			0.7			0.8			1.0			1.2		
Cleaning no	Standard cleanedarea (px)	Cleaning efficacy (%)	Standard error (%)	Standard cleaned area (px)	Cleaning efficacy (%)	Standard error (%)	Standard cleaned area (px)	Cleaning efficacy (%)	Standard error (%)	Standard cleaned area (px)	Cleaning efficacy (%)	Standard error (%)	Standard cleaned area (px)	Cleaning efficacy (%)	Standard error (%)
1	11,720	28.91	2.07	11,890	29.32	1.89	10,631	26.22	2.65	12,101	29.84	0.21	15,647	38.59	1.35
2	17,876	44.09	3.58	17,673	43.59	2.43	18,835	46.45	2.00	18,878	46.56	1.42	21,598	53.27	0.74
3	22,507	55.51	1.83	23,531	58.04	2.78	25,887	63.85	0.49	23,561	58.11	2.04	26,564	65.52	0.10
4	26,840	66.20	0.33	27,694	68.30	1.25	29,584	72.97	0.64	27,572	68.00	2.33	31,454	77.58	0.37
5	28,085	69.27	0.59	28,870	71.20	0.64	30,359	74.88	0.79	30,053	74.12	1.14	33,847	83.48	1.65
6	28,347	69.92	0.99	29,262	72.17	0.22	30,748	75.84	0.91	31,101	76.71	1.74	34,284	84.56	1.64
7	28,787	71.00	0.37	29,456	72.65	0.44	30,984	76.42	0.83	31,714	78.22	0.78	34,662	85.49	1.84
8	29,030	71.60	0.13	29,719	73.30	0.50	31,391	77.42	0.86	32,173	79.35	0.37	34,852	85.96	1.68
9	29,211	72.04	0.43	29,882	73.70	0.60	31,526	77.75	0.76	32,369	79.84	0.39	35,019	86.37	1.66

**Table 2:** Summary of standard cleaned area, cleaning efficacy, and standard error for interdental brushes of six sizes over the cycles from 0 to 9 at 1.2 mm embrasure (n = 5)

Brush stem diameter (mm)	0.6			0.7			0.8			1.0			1.2			1.5		
Cleaning no	Standard cleaned area (px)	Cleaning efficacy (%)	Standard error (%)	Standard cleaned area (px)	Cleaning efficacy (%)	Standard error (%)	Standard cleaned area (px)	Cleaning efficacy (%)	Standard error (%)	Standard cleaned area (px)	Cleaning efficacy (%)	Standard error (%)	Standard cleaned area (px)	Cleaning efficacy (%)	Standard error (%)	Standard cleaned area (px)	Cleaning efficacy (%)	Standard error (%)
1	7808	19.26	0.91	9544	23.54	0.31	11,161	27.53	0.82	12,021	29.65	1.96	14,248	35.14	1.30	18,767	46.29	2.31
2	13,439	33.15	1.56	14,064	34.69	0.85	16,392	40.43	2.08	16,497	40.69	1.46	18,337	45.23	1.71	21,891	53.99	2.53
3	18,051	44.52	0.97	18,506	45.64	0.37	20,259	49.97	1.80	20,579	50.76	2.46	22,385	55.21	1.12	26,516	65.40	2.74
4	19,319	47.65	0.91	21,999	54.26	0.43	23,145	57.09	2.00	24,315	59.97	2.34	25,682	63.34	1.25	28,257	69.69	2.50
5	20,172	49.75	0.61	23,227	57.29	0.60	24,917	61.46	1.14	25,531	62.97	1.27	26,665	65.77	1.13	28,678	70.73	2.40
6	20,592	50.79	0.72	23,621	58.26	0.40	25,531	62.97	0.78	26,726	65.92	0.89	27,539	67.92	1.02	29,577	72.95	1.66
7	20,881	51.50	0.66	23,816	58.74	0.40	26,006	64.14	0.45	27,119	66.89	0.59	27,995	69.05	1.07	29,925	73.81	1.53
8	21,164	52.20	0.30	24,034	59.28	0.62	26,093	64.36	0.44	27,565	67.99	0.80	28,344	69.91	0.78	30,299	74.73	1.56
9	21,280	52.48	0.26	24,245	59.80	0.63	26,240	64.72	0.49	27,738	68.41	0.67	28,633	70.62	0.41	30,569	75.39	1.45

## Discussion

Hygienic maintenance of the interdental space is encouraged to prevent periodontal diseases [20, 21]. With the help of 3D printing technology, it was possible to quantify the cleaning efficacy of the interdental brush on the embrasure surfaces of the teeth. Most notably, while the cleaning efficacy was positively correlated with the number of washes, a plateau was obtained starting from the 5th cycle. Moreover, it was confirmed that there was a positive correlation between the diameter of the IDBs and cleaning efficacy. This evidence supported the previous article’s claim that the size and specifications of oral hygiene aids such as IDBs should be accurately selected [22, 23]. Herein, the plateau obtained after the 5th washing cycle shows the same tendency in other studies [11]. Contrary to some prior research that showed that the cleaning effect was greater as the number of cleaning cycles increased, the results of this study could be a guideline for practical use [11]. Repeated cleaning does not need to be for more than five cycles because there is a higher risk of interdental gingival (papillary) bleeding, which can add to adverse effects in addition to the cleaning [2].

From what we have found, the IDB select guide line recommends fit [24] or slightly larger [9] size for IDR, and should consider force [22] as well as shape and bristles of IDB. Since tooth abrasion cannot be avoided and root surface with dentin is weaker than enamel, the appropriate number of cycles should be selected to reduce as much damage as possible. Therefore, we made a model base (gum) for constant and accurate moving test so that so that teeth can be inserted, removed, and controlled. The model itself was fixed, elbow rest was given at a certain position, and positioning was performed so that only the reciprocating motion could be performed. In this study, IDB of a size greater than the given embrasure gave the optimal performance diameters of 1.2 and 1.5 mm at 0.7 and 1.2 mm embrasures, respectively. A previous study presented the 10th cycle on the molar using dental floss, holder-type dental floss, and IDBs (size SSS) and observed the mesial side [25]. In this study, the cleaning efficacy was shown in the order of floss (53%), IDBs (46%), and floss holder (28%), but since the embrasure size was not specified, it was somewhat difficult to interpret this as a quantitative experimental result. Meanwhile, dental experts suggest using IDBs of sizes that are slightly larger than the patient’s embrasure size, and this study supports the experts’ hypotheses. This is thought to be because the toothbrush has to be longer than the actual diameter to penetrate deeper as the shape of the actual tooth is concave. The cleaning efficacy of an IDB is influenced by the design and diameter of the IDB, and the size of the interdental space [26, 27]. Therefore, further studies are needed on the effect of these factors like varying diameters and shapes of IDBs on cleaning efficacy.

Additionally, in this study, the experiments were conducted with interdental space and various embrasure sizes that were customized in proportion to the actual human physique. In previous clinical research, patients were made to use IDBs for three months to compare the effectiveness of conically shaped and cylindrically shaped IDBs; and plaque scores, bleeding upon pocket probing scores, and probing pocket depth were assessed [24]. Though the patients were educated by specialists who worked on the experiments, apparently, the reproducibility was reduced for each patient. Furthermore, there was the limitation that the evaluation criteria lacked objectivity. 3D printing technology is widely adopted in the field of dentistry, for example, crowns, bridges, reconstructors, splints, and implants [12]. Previous research investigated the high accuracy of 3D-printed teeth [28]. However, to the best of our knowledge, a study used a 3D-printer to determine human teeth and match morphologically equivalent pairs for different spacing sizes using rubber picks. But no study focused on evaluation of the IDBs in different sizes using the 3D-print model. However, there was a study that tested cylindrical IDBs (0.8, 0.9, 1.2, and 1.4 mm) and interdental tooth surfaces constructed by a 3D printer based on human teeth and matched to morphologically equivalent pairs, such as the isosceles triangle, concave and convex fitting to the different gap sizes (1.0, 1.1, and 1.3 mm) [23]. In an isosceles triangle with a 1 mm gap, IDBs with a 0.9 mm diameter showed the highest cleaning efficiency at 84%. Thus, IDBs can be tested by a new experimental setup supported by 3D printing technology. Moreover, it is possible to conduct more direct and definite experiments through 3D printing by designing various human interdental spaces and embrasures with CAD software. This study will serve as a benchmark for conducting experiments by grafting not only IDBs but also various oral products.

This testification provides an example of the delicate modeling and manufacturing of such anatomical 3D architecture performed expeditiously and conveniently. The current model provides a fundamental solution to and suggestion for one of the largest unmet needs of patients and practitioners systemically. However, there are many limitations to our study. First, actual dental plaque is different from an artificial plaque in its characteristics. Second, there are differences between the 3D-printed oral structure and the actual biometric oral structure. Third, cleaning efficacy according to the shape of the IDB was not considered. Fourth, the morphology of the embrasure was implemented with only one shape. However, the basic research was conducted; and the observation of quantitative measures on cleaning efficiency was attempted [10, 29]. As the shape of the IDB affects the cleaning efficacy, additional research must identify its correlation with various IDB specifications.

## Conclusions

In this study, we demonstrated that the 3D-printed model base for the human oral embrasure structure is an adequate model to affirm artificial plaque removal using the IDB. A major strength of the 3D-printed model base is that the customization of the embrasure sizes in a reproducible manner was enabled by the 3D model designed on the CAD software. With the repetitive move cycles of the IDBs of different sizes, we observed the efficacy reaching a plateau state and could find that the optimal diameter for the IDB was at 1.2 and 1.5 mm for embrasure sized at 0.7 and 1.2 mm, respectively. This report provides a fundamental guideline when selecting the size of the IDB for a given embrasure gap.

## Data Availability

The datasets used and/or analyzed during the current study are available from the corresponding author on reasonable request.

## Abbreviations

ICAs: Interdental cleaning aids
IDBs: Interdental brushes
CAD/CAM: Dental computer-aided design/computer-aided manufacturing
